# Case Report: ^18^F-FDG PET/CT revealed diffuse pancreatic metastasis in paranasal sinuses rhabdomyosarcoma after treatment improvement

**DOI:** 10.3389/fmed.2025.1609837

**Published:** 2025-05-30

**Authors:** Xianwen Hu, Haojun Duan, Ying Zhao, Yongpeng Wang, Dongfeng Pan

**Affiliations:** ^1^Department of Nuclear Medicine, Affiliated Hospital of Zunyi Medical University, Zunyi, China; ^2^The Second Clinical College, Dalian Medical University, Dalian, China; ^3^Department of Nuclear Medicine, The Second Affiliated Hospital of Zunyi Medical University, Zunyi, China

**Keywords:** rhabdomyosarcoma, pancreatic metastasis, ^18^F-FDG, PET/CT, autoimmune pancreatitis

## Abstract

Metastasis of rhabdomyosarcoma (RMS) from primary paranasal sinuses to the pancreas is rare. Herein, we present a rare case of diffuse pancreatic metastasis in a 44-year-old man. He was diagnosed with sinus RMS 1 year ago and the lesion disappeared after systematic treatment. The patient came to our hospital in November 2022 due to abdominal pain. Abdominal ultrasound showed diffuse enlargement of the pancreas, with enlarged lymph nodes visible around the pancreas and retroperitoneum. He then underwent positron emission tomography/computed tomography (PET/CT) imaging and the results showed that the pancreas had diffusely increased fluorine-18 fluorodeoxyglucose (^18^F-FDG) uptake, and the surrounding pancreas and retroperitoneal lymph nodes also had increased ^18^F-FDG uptake, suggesting a possibility of malignant tumor. Ultimately, a pancreatic puncture biopsy confirmed RMS with pancreatic and abdominal lymph nodes metastasis. Our case suggests that although diffuse metastatic tumors of the pancreas are relatively rare, which should be considered as one of the differential diagnoses for diffuse pancreatic hypermetabolic lesions on PET/CT imaging.

## Introduction

Rhabdomyosarcoma (RMS) is a soft tissue malignancy originating from immature mesenchymal cells that have the potential to differentiate into striated muscle (1). The tumor is more common in children and adolescents while less common in adults (2). It can develop in diverse anatomical sites, including striated muscle-rich areas such as the head and neck and limb soft tissues, as well as regions that lack striated muscles including bladder, uterus and so on (3). Metastasis sites are mostly lung, bone and lymph nodes, while metastasis to pancreas, especially diffuse metastasis, is rarely reported in the literature. Metabolic activity is assessed with both PET and CT-fused images, and has unique value in monitoring RMS staging and recurrence (4). This paper reports a rare case of diffuse pancreatic metastasis‌ revealed by ^‌18^F-FDG [positron emission tomography/computed tomography (PET/CT)‌] in a patient with ‌sinonasal RMS‌ who developed abdominal pain ‌2 years after initial treatment‌, and discusses the diagnostic challenges associated with such cases.

## Case presentation

**Figure 1:**
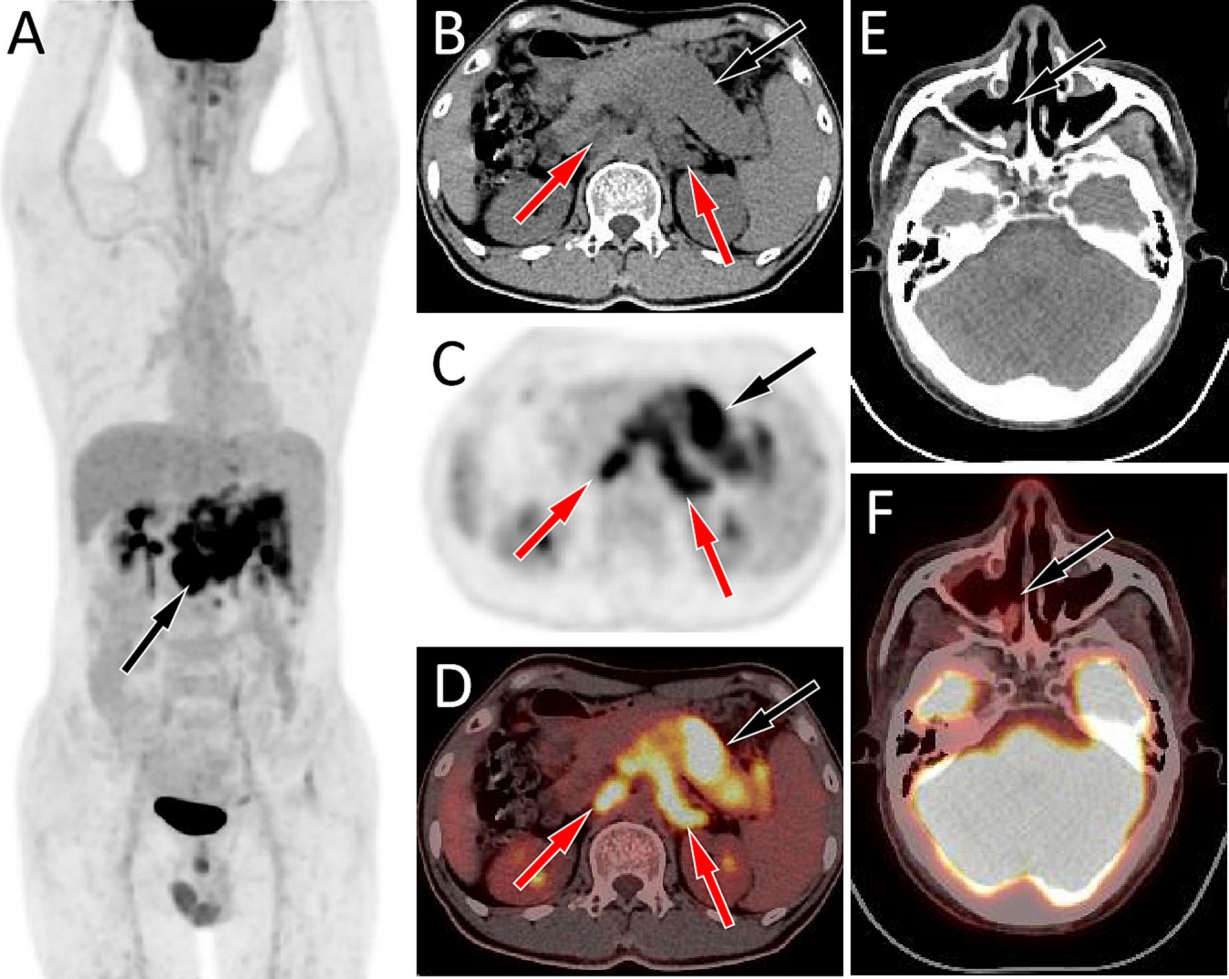
Fluorine-18 fluorodeoxyglucose (^18^F-FDG) positron emission tomography (PET)/CT imaging of the patient; The maximum intensity projection (MIP, **A**) showed a slightly increased ^18^F-FDG uptake in the upper abdomen (arrow). Axial CT **(B)** showed diffuse enlargement of the pancreas, mainly in the body and tail regions, presenting as slightly low density (black arrow), and multiple enlarged lymph nodes are observed around the pancreas and in the retroperitoneum (red arrows). The corresponding lesions had obviously increased ^18^F-FDG uptake on axial PET **(C)** and PET/CT fusion **(D)**, with the maximum standardized uptake values (SUVmax) being 10.8 and 11.7, respectively. However, no obvious soft tissue mass on CT (**E**, arrow) and increased ^18^F-FDG uptake on PET/CT (**F**, arrow) was observed in the right maxillary sinus area.

**Figure 2:**
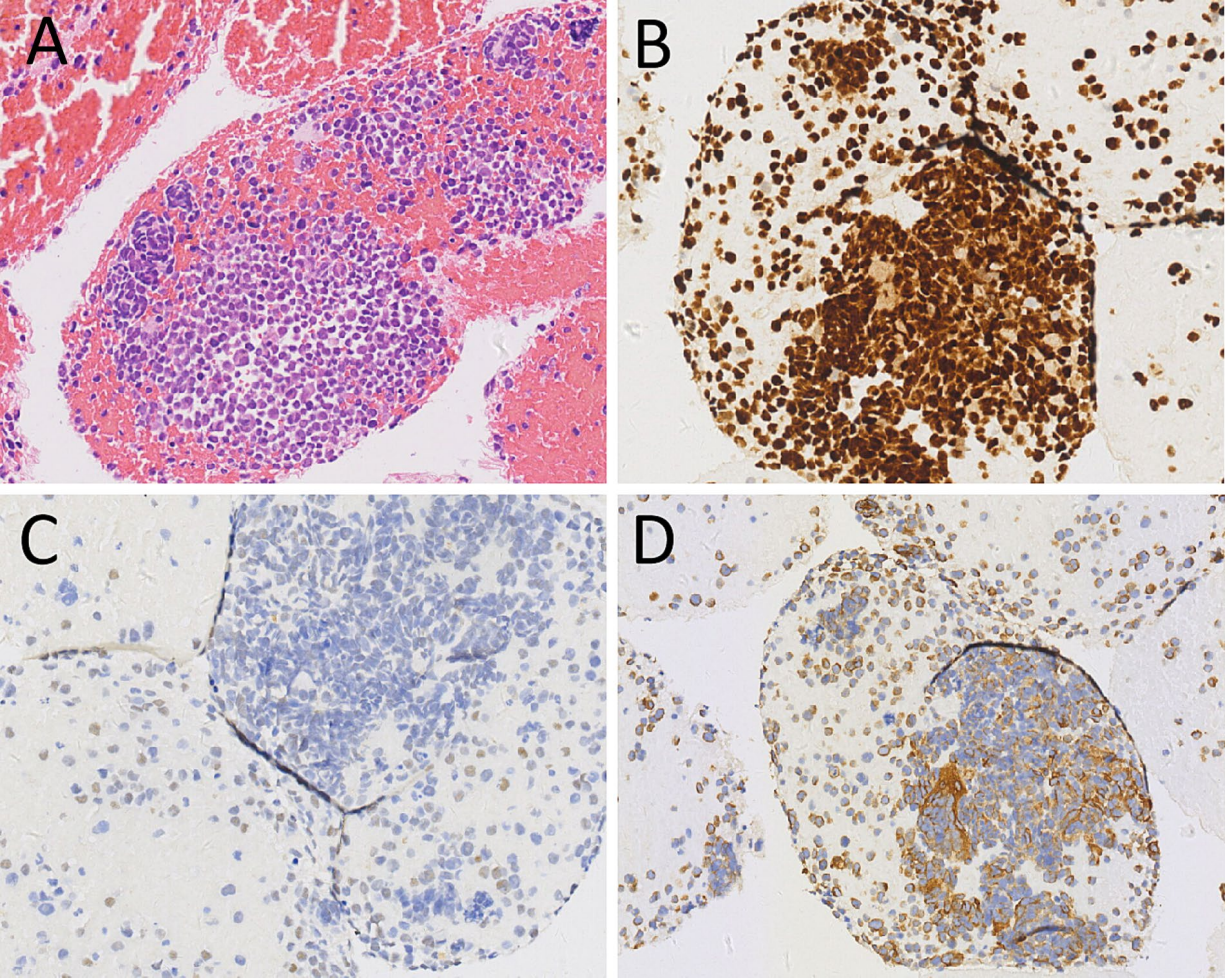
Hematoxylin–eosin staining **(A)** showed scattered pleomorphic cells, multinucleated cells and monocytes in the tumor tissue. Immunohistochemical staining revealed that the tumor cells positively expressed Myogenin **(B)**, MyoD1 **(C)** and Desmin **(D)**. These pathological histological features and immunohistochemical results support the diagnosis of rhabdomyosarcoma.

A 44-year-old male patient presented to our hospital in November 2022 due to persistent abdominal and low back pain for 1 month. He was diagnosed with RMS of the right maxillary sinus and ethmoid sinus by endoscopy in an external hospital in March 2021, followed by endoscopic fenestration of the right maxillary sinus and ethmoid sinus, and 5 courses of isocyphosphamide-etoposide (IE) regimen chemotherapy. Magnetic resonance imaging (MRI) evaluation in September 2021 after the above treatment revealed complete remission of the right maxillary sinus and ethmoid sinus lesions. His family had no history of cancer. Physical examination revealed obvious abdominal periumbilical tenderness. Serum oncology markers including Ca199, carcinoembryonic antigen (CEA), and Ca125 were negative. Abdominal ultrasound revealed diffuse enlargement of his pancreas, evident in the body and tail, with enlarged lymph nodes around the pancreatic head and retroperitoneum. Subsequently, the patient underwent PET/CT to further evaluate the nature of the lesion. The results showed diffuse metabolic elevation in the patient’s pancreas, with multiple metabolic elevation lymph nodes visible around the pancreatic head and retroperitoneum (as shown in Figure 1), suggesting the possibility of malignant tumors; Nevertheless, there were no signs of tumor recurrence or residual tumor in the right maxillary sinus and right ethmoid sinus. Routine histopathological microscopic examination demonstrated that the lesional tissue acquired by puncture was predominantly composed of cells with variable sizes, primarily round in shape, and a minority displaying short spindle-shaped or ovoid morphology. These cells exhibited small nuclei with hyperchromatic staining and sparse cytoplasm showing pale eosinophilic reddish coloration. Immunohistochemical staining demonstrated positive expression of Desmin, Myogenic Differentiation 1 (MyoD1), and Myogenin in the tumor cells (Figure 2), with a Ki-67 proliferation index of 70%, while were negative for CD20, cytokeratin (CK), CD79a, CD38, and chromogranin A (CgA). Based on the patient’s tumor history and histopathological findings, RMS metastasis to pancreatic and abdominal lymph nodes was confirmed. After diagnosis, the patient received a chemotherapy regimen of ifosfamide (3 g IV d1-d5) and epirubicin (100 mg IV d1-d2), during which grade IV bone marrow suppression occurred, which was relieved after symptomatic treatment. After 8 courses of treatment according to the above scheme, adjust the dosage (oral alotinib 12 mg combined with epirubicin 100 mg d1), and then carry out standard two courses of chemotherapy.

**Figure 3:**
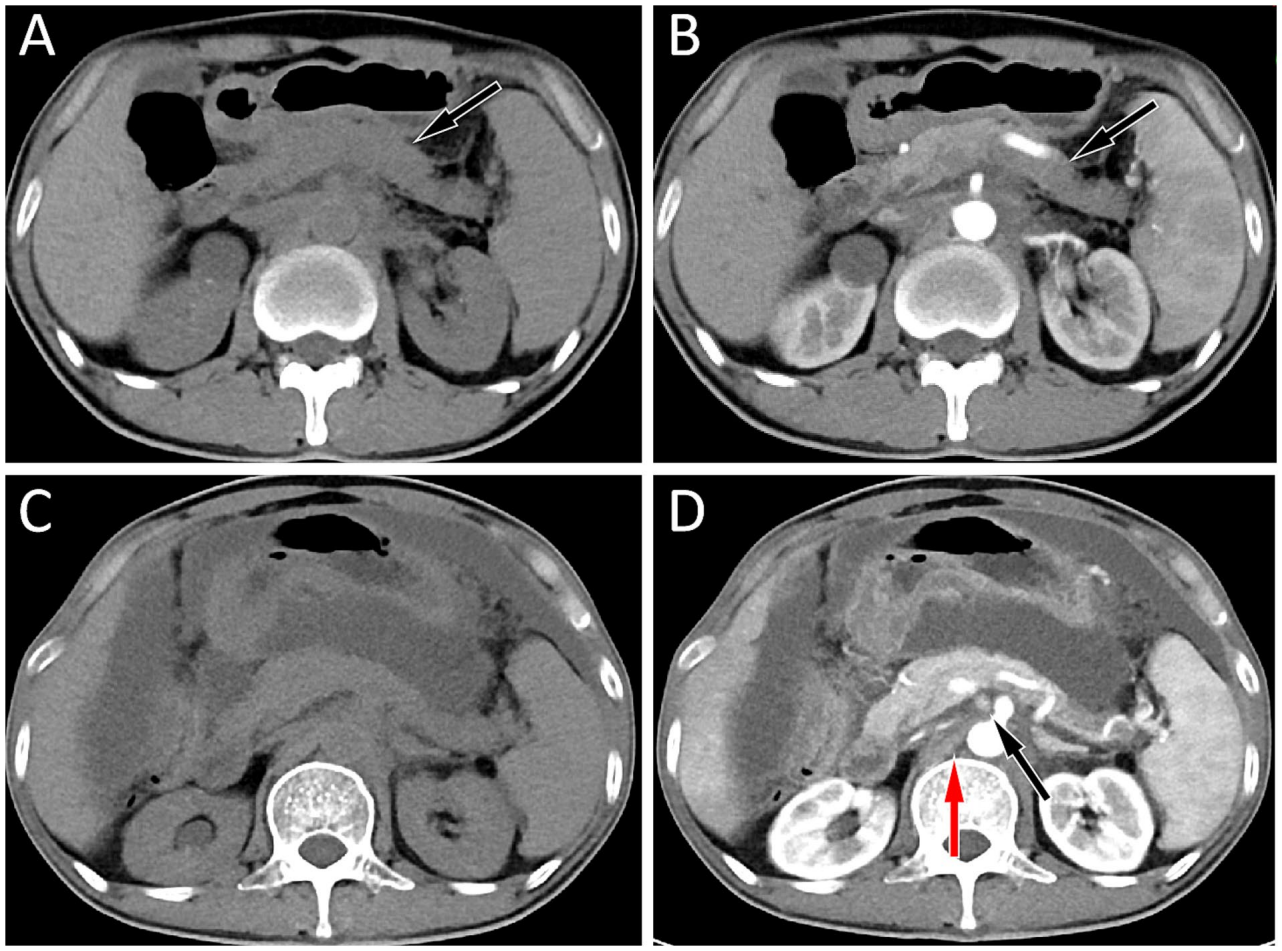
**(A,B)** The abdominal computed tomography (CT) scan of the patient in May 2023, and the results showed a decrease in pancreatic volume and disappearance of enlarged lymph nodes around the pancreas and retroperitoneum in both plain **(A)** and contrast-enhanced CT **(B)** scans; **(C,D)** His abdominal CT in July 2023, plain scan **(C)** and contrast-enhanced CT **(D)** revealed a large amount of fluid in the abdominal cavity, and multiple enlarged lymph nodes were seen around the pancreas (black arrow) and in the retroperitoneum (red arrow), suggesting tumor recurrence.

Subsequently, the patient underwent abdominal CT examination in 2023. The result showed that the volume of the pancreas had shrunk, and the lymph nodes around the pancreas and in the retroperitoneum were displayed, suggesting that the condition had improved. However, 2 months later, the patient again presented with symptoms of abdominal distension and abdominal pain. The abdominal CT scan (as shown in Figure 3) indicated that there was a large amount of ascites in the abdominal cavity, and enlarged lymph nodes appeared around the pancreas and in the retroperitoneum, suggesting a possible recurrence of the tumor. The patient received chemotherapy regimens of ifosfamide and epirubicin again, but unfortunately, his condition deteriorated further and he eventually passed away in September 2023.

## Discussion

Pancreatic metastases are the manifestation of advanced tumors and are relatively rare in clinical practice, with an incidence of only 2 to 5% (5, 6). The most common tumors with pancreatic metastasis are lung cancer, breast cancer, stomach cancer, kidney cancer, melanoma, colorectal cancer, etc., of which lung cancer accounts for more than 60% of all pancreatic metastases (7). However, RMS metastasis to the pancreas is rare. The possible mechanism of RMS metastasis to the pancreas is that tumor cells enter the liver through the portal vein via blood circulation, and then reflux to the pancreas through the hepatic vein, forming secondary metastases; Or tumor cells may metastasize to adjacent lymph nodes (such as the abdominal aorta and pancreatic lymph nodes) through the lymphatic network, and then invade the surrounding tissues of the pancreas along the lymphatic chain (8). The primary tumor of the current case we presented was paranasal sinus RMS, whose metastasis to the pancreas was rarely reported in the literature.

Pancreatic metastases may be single nodules or multiple nodules, but diffuse metastases are rare. The plain CT scan density of pancreatic metastases is mostly equal density or slightly low density, and the tumors are often accompanied by low-density necrotic areas, but calcification is rare, which showed mostly mild uneven enhancement, mainly edge enhancement on contrast-enhanced CT (7). Pancreatic diffuse metastatic tumors have a high malignant potential and typically present as increased diffuse ^18^F-FDG uptake in the pancreas on PET, often accompanied by enlargement of lymph nodes around the pancreas and in the retroperitoneum, which also show increased ^18^F-FDG uptake (9). The patient we are currently reporting presents with enlarged pancreatic volume on CT, mostly with equal soft tissue density and slightly low-density necrotic areas within it. On PET, there is diffuse increased ^18^F-FDG uptake, and enlarged lymph nodes with increased glucose metabolism around the pancreas and retroperitoneum, which is consistent with the imaging findings of diffuse metastatic tumors of the pancreas reported above.

The imaging findings of diffuse pancreatic metastasis should be differentiated from other diffuse pancreatic diseases such as autoimmune pancreatitis (AIP), pancreatic lymphoma, pancreatic cancer with obstructive pancreatitis. AIP has a low incidence rate and can be divided into two types: type 1 is lymphoplasmacytic sclerosing pancreatitis, which is closely related to IgG4, that is, IgG4 related AIP; Type 2 is idiopathic catheter-related chronic pancreatitis. AIP showed “sausage-like” or “chain-like” changes on CT, without hemorrhage and necrosis, less pancreatic duct dilation, no invasion or embedding changes in peripancreatic blood vessels, and the peripancreatic fat space was clear, which was different from pancreatic malignant tumors (10). PET/CT whole-body imaging can show the morphological changes and changes in glucose metabolism of the diseased pancreatic tissues, which are typically presented as increased diffuse ^18^F-FDG uptake, with a maximum standardized uptake value (SUVmax) of 7.0 ± 2.3 (range, 4.0–10.2), but usually lower than SUVmax of malignant pancreatic tumors (11). Moreover, PET/CT can also show the involvement of external organs of the pancreas such as internal and external hepatic bile duct, salivary glands, lymph nodes, etc., so it has been widely used in the diagnosis and differential diagnosis of AIP (12, 13). Pancreatic lymphoma is mostly secondary, with primary pancreatic lymphoma accounting for less than 5% of pancreatic malignancies, and the pathological type is mostly diffuse large B-cell lymphoma (14). On CT, it presents as homogeneous or nodular enlargement of the pancreas, with uniform density of the mass, while cystic degeneration, necrosis, or calcification are rare, and pancreatic ductal dilatation is uncommon. The mass often surrounds surrounding vessels such as the superior mesenteric artery/vein and portal vein, but rarely causes vascular invasion (15). Lymphoma cells are metabolically active, and they are presented on PET as a significantly increasd in the ^18^F-FDG uptake in the lesion area, of which SUVmax is usually higher than that of pancreatic cancer and pancreatitis; moreover, PET/CT imaging can also detect lymph nodes and other organs and tissues involved throughout the body (16). Pancreatic cancer is a common malignant tumor of the pancreas. When pancreatic cancer obstructs the distal common bile duct or pancreatic duct, it can lead to obstructive inflammation of the pancreas, which results in diffuse increased ^18^F-FDG uptake in the pancreas on PET/CT, with a SUVmax comparable to that of pancreatic metastases (17). However, different from pancreatic metastases, pancreatic cancer often causes bile duct or pancreatic duct dilatation. When accompanied with obstructive pancreatitis, peripancreatic exudation is more obvious, and pancreatic metastases usually have a history of primary tumors.

Diagnosis of pancreatic metastases is confirmed by histopathological examination, and their histopathological features are identical to those of the primary tumor. The characteristics of RMS include diverse morphology of tumor cells under the microscope, sparse cytoplasm, usually small nuclei, deep staining, easily visible nuclear division, spindle shaped and strip-shaped cytoplasm, and striped cytoplasm, indicating skeletal muscle differentiation (18). When RMS is suspected, further immunohistochemical examination can be performed. RMS usually expresses the rhabdomyolysis related antigens such as Myogenin, MyoD1, Desmin, but does not express CgA, Syn, CD2, CD5, CD19, etc. (19). The case we reported showed scattered pleomorphic cells, multinucleated cells and monocytes in the tumor tissue under the microscope, with loose cell adherents, eccentric nuclei, abnormal nuclear membranes, large and malformed cells, and the cytoplasm was spindle shaped and zoned. Immunohistochemical staining revealed positive expression of Myogenin, MyoD1 and Desmin in the tumor cells. These features are consistent with the diagnosis of RMS.

When RMS metastases to the pancreas, it is usually accompanied by metastasis to other sites, so it can only receive palliative treatment such as chemotherapy and radiotherapy, and its prognosis is poor, and its 5-year survival rate is less than 30% (20). The patient we are currently reporting experienced distant metastasis within less than a year of initial diagnosis of RMS. Despite receiving systematic treatment, unfortunately, the patient passed away in less than 2 years, revealing the highly malignant nature and poor prognosis of RMS.

## Conclusion

Metastasis of primary paranasal sinus RMS to the pancreas is rare, and PET/CT is important in the evaluation after RMS treatment. When the pancreas shows an increased diffuse ^18^F-FDG uptake, metastatic tumors should be considered as one of the differential diagnoses of diffuse pancreatic lesions.

## Data Availability

The original contributions presented in the study are included in the article/supplementary material, further inquiries can be directed to the corresponding authors.

## References

[1] Patrichi AI Gurzu S. Pathogenetic and molecular classifications of soft tissue and bone tumors: a 2024 update. Pathol Res Pract. (2024) 260:155406. doi: 10.1016/j.prp.2024.155406, PMID: 38878666

[2] Arndt CA Rose PS Folpe AL Laack NN. Common musculoskeletal tumors of childhood and adolescence. Mayo Clin Proc. (2012) 87:475–87. doi: 10.1016/j.mayocp.2012.01.015, PMID: 22560526 PMC3538469

[3] Leiner J Le Loarer F. The current landscape of rhabdomyosarcomas: an update. Virchows Arch. (2020) 476:97–108. doi: 10.1007/s00428-019-02676-9, PMID: 31696361

[4] Vaarwerk B Breunis WB Haveman LM de Keizer B Jehanno N Borgwardt L. Fluorine-18-fluorodeoxyglucose (FDG) positron emission tomography (PET) computed tomography (CT) for the detection of bone, lung, and lymph node metastases in rhabdomyosarcoma. Cochrane Database Syst Rev. (2021) 2021:CD012325. doi: 10.1002/14651858.CD012325.pub2, PMID: 34753195 PMC8577863

[5] Geramizadeh B Kashkooe A Nikeghbalian S Malek-Hosseini SA. Metastatic tumors to the pancreas, a single center study. Arch Iran Med. (2019) 22:50–2. PMID: 30821161

[6] Nakajima Y Iwasaki E Kayashima A Machida Y Kawasaki S Horibe M. Successful radiotherapy for recurrent obstructive pancreatitis secondary to pancreatic metastasis from cervical squamous-cell carcinoma. Clin J Gastroenterol. (2023) 16:755–60. doi: 10.1007/s12328-023-01817-7, PMID: 37269479

[7] Okauchi S Miyazaki K Shiozawa T Satoh H Hizawa N. Distant organ metastasis patterns in lung cancer patients with pancreatic metastasis - a cluster analysis. Contemp Oncol. (2022) 26:247–51. doi: 10.5114/wo.2022.120593, PMID: 36381670 PMC9641633

[8] Sekulic M Amin K Mettler T Miller LK Mallery S Rd SJ. Pancreatic involvement by metastasizing neoplasms as determined by endoscopic ultrasound-guided fine needle aspiration: a clinicopathologic characterization. Diagn Cytopathol. (2017) 45:418–25. doi: 10.1002/dc.23688, PMID: 28205397

[9] Wang XY Yang F Jin C Fu DL. Utility of PET/CT in diagnosis, staging, assessment of resectability and metabolic response of pancreatic cancer. World J Gastroenterol. (2014) 20:15580–9. doi: 10.3748/wjg.v20.i42.15580, PMID: 25400441 PMC4229522

[10] Takahashi H Yamashita H Morooka M Kubota K Takahashi Y Kaneko H. The utility of FDG-PET/CT and other imaging techniques in the evaluation of IgG4-related disease. Joint Bone Spine. (2014) 81:331–6. doi: 10.1016/j.jbspin.2014.01.010, PMID: 24568886

[11] Bohl CE Federico SM Robinson GW Bahrami A Shulkin BL. FDG-PET CT in the evaluation of primary and secondary pancreatic malignancies. Pediatr Blood Cancer. (2018) 65:e27115. doi: 10.1002/pbc.27115, PMID: 29750397

[12] Datta D Selvakumar B Goel AD Chhibber S Varshney VK Kumar R. Diagnostic performance of F-18 FDG PET/CT in differentiating autoimmune pancreatitis from pancreatic cancer: a systemic review and meta-analysis. Ann Nucl Med. (2024) 38:619–29. doi: 10.1007/s12149-024-01934-4, PMID: 38750330

[13] Ohtani M Ofuji K Akazawa Y Saito Y Nosaka T Ozaki Y. Clinical usefulness of [18F]-Fluoro-2-Deoxy-d-glucose-positron emission tomography/computed tomography for distinguishing between autoimmune pancreatitis and pancreatic Cancer. Pancreas. (2021) 50:1014–9. doi: 10.1097/MPA.0000000000001873, PMID: 34629452

[14] Anand D Lall C Bhosale P Ganeshan D Qayyum A. Current update on primary pancreatic lymphoma. Abdom Radiol. (2016) 41:347–55. doi: 10.1007/s00261-015-0620-8, PMID: 26830418

[15] Merkle EM Bender GN Brambs HJ. Imaging findings in pancreatic lymphoma: differential aspects. AJR Am J Roentgenol. (2000) 174:671–5. doi: 10.2214/ajr.174.3.1740671, PMID: 10701607

[16] Pode D Lenkovsky Z Shapiro A Pfau A. Can extracorporeal shock wave lithotripsy eradicate persistent urinary infection associated with infected stones. J Urol. (1988) 140:257–9. doi: 10.1016/s0022-5347(17)41577-1, PMID: 3398117

[17] Carlson DM Abdelrahman AM Adjei Antwi SK Tomlinson JL Trivedi K Karbhari A. Baseline characteristics and use of Pretherapeutic 18 F-Fluorodeoxyglucose-PET for pancreatic Cancer. J Am Coll Surg. (2024) 239:9–17. doi: 10.1097/XCS.0000000000001059, PMID: 38445645 PMC11168783

[18] Rudzinski ER Teot LA Anderson JR Moore J Bridge JA Barr FG. Dense pattern of embryonal rhabdomyosarcoma, a lesion easily confused with alveolar rhabdomyosarcoma: a report from the soft tissue sarcoma Committee of the Children's oncology group. Am J Clin Pathol. (2013) 140:82–90. doi: 10.1309/AJCPA1WN7ARPCMKQ, PMID: 23765537 PMC4624292

[19] Anderson WJ Doyle LA. Updates from the 2020 World Health Organization classification of soft tissue and bone Tumours. Histopathology. (2021) 78:644–57. doi: 10.1111/his.14265, PMID: 33438273

[20] Jha P Frölich AM McCarville B Navarro OM Babyn P Goldsby R. Unusual association of alveolar rhabdomyosarcoma with pancreatic metastasis: emerging role of PET-CT in tumor staging. Pediatr Radiol. (2010) 40:1380–6. doi: 10.1007/s00247-010-1572-3, PMID: 20180103 PMC2895865

